# Inflammasomes in Atherosclerosis—From Pathophysiology to Treatment

**DOI:** 10.3390/ph16091211

**Published:** 2023-08-28

**Authors:** Panagiotis Theofilis, Evangelos Oikonomou, Christos Chasikidis, Konstantinos Tsioufis, Dimitris Tousoulis

**Affiliations:** 1 1st Department of Cardiology, “Hippokration” General Hospital, National and Kapodistrian University of Athens, 11527 Athens, Greece; panos.theofilis@hotmail.com (P.T.);; 2 3rd Department of Cardiology, Thoracic Diseases General Hospital “Sotiria”, National and Kapodistrian University of Athens, 11527 Athens, Greece; 3 Department of Cardiology, General Hospital of Corinth, 20100 Corinth, Greece

**Keywords:** NLRP3 inflammasome, inflammation, atherosclerosis

## Abstract

Atherosclerosis, a chronic inflammatory disease characterized by arterial plaque accumulation, remains a significant global health challenge. In recent years, inflammasomes, the intracellular multiprotein complexes crucial for initiating innate immune responses, have emerged as key players in atherosclerosis pathophysiology. This review article aims to provide a comprehensive overview of the current understanding of inflammasome activation and its impact on atherosclerosis development and progression. We explore the intricate interplay between traditional cardiovascular risk factors and inflammasome activation, leading to the perpetuation of inflammatory cascades that drive plaque formation and instability. The review focuses on the molecular mechanisms underlying inflammasome activation, including the role of pattern recognition receptors and cytokines in this process. Moreover, we discuss the contribution of inflammasomes to endothelial dysfunction, foam cell formation, and vascular inflammation. Additionally, recent advances in therapeutic strategies targeting inflammasomes are examined, including pharmacological agents and potential immunomodulatory approaches. By collating and analyzing the current evidence, this review provides valuable insights into the potential of inflammasome-targeted therapies for atherosclerosis management and treatment. Understanding the pivotal role of inflammasomes in atherosclerosis pathophysiology offers promising prospects for developing effective and personalized therapeutic interventions that can mitigate the burden of this prevalent cardiovascular disorder and improve patient outcomes.

## 1. Introduction

Atherosclerosis, characterized by the accumulation of lipid-rich plaques within arterial walls, is a chronic progressive vascular condition resulting in narrowed arterial lumen and, ultimately, major cardiovascular events such as myocardial infarction, ischemic stroke, or critical limb ischemia [1]. It has been widely considered as the leading cause of morbidity and mortality worldwide [2,3], imposing an immense burden on healthcare systems. Despite extensive research, the intricacies of atherosclerotic development and progression have proven to be multifaceted and involve complex interactions between various cell types, inflammatory mediators, and genetic factors [4,5]. In recent years, the recognition of the crucial role played by inflammasomes in the pathophysiology of atherosclerosis has emerged as a promising avenue for novel therapeutic interventions [6].

Inflammasomes represent intracellular multi-protein complexes responsible for initiating the innate immune response by activating caspase-1, which ultimately leads to the production and release of proinflammatory cytokines such as interleukin-1β (IL-1β) and interleukin-18 (IL-18). Mounting evidence suggests that these inflammasome-mediated inflammatory responses play a pivotal role in driving the initiation, propagation, and complication of atherosclerotic plaques, thereby exacerbating disease severity [5].

Given the pivotal role of inflammasomes in atherosclerosis pathogenesis, researchers and clinicians have shifted their focus toward understanding the molecular mechanisms underlying inflammasome activation and its downstream effects [7]. Additionally, exploiting this knowledge to develop targeted therapies that modulate inflammasome activity has gained significant attention as a potential means of halting disease progression and reducing atherosclerosis-associated complications [5].

This review article aims to comprehensively explore the current understanding of the involvement of inflammasomes in atherosclerosis pathophysiology, with a focus on the cellular and molecular mechanisms driving their activation. Moreover, we will delve into the recent advancements in therapeutic strategies targeting mainly NLRP3 inflammasome, including existing anti-atherosclerotic pharmacotherapy with proven NLRP3 inflammasome-modulating effects, direct NLRP3 inflammasome inhibitors, and agents with secondary NLRP3 inflammasome inhibitory effects. Furthermore, we will discuss the available evidence concerning the modulation of other inflammasomes in atherosclerosis research. By collating the existing evidence, we seek to offer insights into the promising clinical implications and future perspectives of inflammasome-targeted therapies for treating atherosclerosis and its associated cardiovascular sequelae, mentioning the key points and challenges that ought to be addressed.

## 2. Atherosclerosis: Pathogenesis and the Role of Inflammation

The atherosclerotic process consists of multiple steps, namely endothelial dysfunction, abnormal lipid metabolism and deposition in the subendothelial layer, and inflammation [8]. Several risk factors contribute to this hazardous cascade, such as arterial hypertension, diabetes mellitus, dyslipidemia, and smoking, among others [1].

The endothelial layer, an abundant organ lining the blood vessels, is crucial in vascular homeostasis, mainly depending on nitric oxide (NO) bioavailability. NO not only regulates vascular tone, but participates in other processes such as thrombosis, fibrinolysis, vascular inflammation, and remodeling [9]. In the setting of impaired NO bioavailability—either due to reduced production or increased degradation—endothelial dysfunction occurs and is considered the initial step of atherogenesis [9]. In such a deleterious state, there is evident vasoconstriction, thrombosis, leukocyte migration, and vascular smooth muscle cell (VSMC) proliferation [9]. Inflammation is crucial in the development of endothelial dysfunction, since proinflammatory cytokines (ILs and tumor necrosis factor-alpha (TNF-α)) can act as damage-associated molecular patterns and stimulate the corresponding endothelial cell receptors, ultimately leading to nuclear factor-κB (NF-κB) signaling [9]. Consequently, there is an upregulation of adhesion molecules (vascular cell adhesion molecule, intercellular adhesion molecule, E-selectin, and P-selectin), which, in turn, aid leukocyte trafficking in the subendothelial layer [9]. Moreover, damaged endothelial cells participate in a vicious cycle of cytokine production that perpetuates the inflammatory cycle [9].

Endothelial impairment additionally facilitates the invasion of apolipoprotein-B-containing lipoproteins, such as low-density lipoproteins (LDL), in the subendothelial layer. Of the known subcategories of LDL, small–dense LDL (sdLDL) is considered the molecule with the greatest atherogenic potential. LDLs are then retained in the arterial wall and undergo several modifications, with oxidation being the most well studied [10]. Foam cell formation ensues as a result of oxidized LDL (oxLDL) binding to the surface lectin-like oxLDL receptor-1 of VSMCs [11], as well as their uptake by macrophages.

## 3. Inflammasomes: A Brief Overview

Although the above-mentioned concepts have been explored in depth, the diverse role of inflammation and the presence of novel inflammatory mediators is under continuous investigation. Among the major elements of the inflammatory process that are relevant to atherosclerosis initiation and progression are the inflammasomes. Inflammasomes are multiprotein complexes that play an important role in the innate immune response, notably in pathogen identification and response, as well as in sterile inflammatory instances [12]. They detect cellular stress, infection, and tissue injury. Inflammasomes are mostly expressed in immune cells such as macrophages, dendritic cells, and neutrophils, but they can also be found in epithelial and endothelial cells [9,13,14,15].

A sensor protein, an adapter protein, and an effector protein are the three major components of an inflammasome [16]. Sensor proteins are in charge of sensing certain pathogen-associated molecular patterns (PAMPs) or danger-associated molecular patterns (DAMPs) and triggering the inflammasome complex’s construction [16]. Sensor proteins such as nucleotide-binding oligomerization domain, leucine-rich repeat and pyrin domain (PYD) containing (NLRP)1, NLRP3, NLR family caspase recruitment domain (CARD)-containing protein (NLRC)4, absent in melanoma (AIM)2, and pyrin are such examples [16]. Each sensor protein has ligand recognition and oligomerization domains. Adaptor proteins operate as a link between the sensor and effector proteins. Apoptosis-associated speck-like protein containing a CARD (ASC), commonly referred to as PYCARD, is the most well-known adaptor protein [16]. ASC features a CARD that interacts with the sensor protein’s CARD domain to facilitate inflammasome formation [16].

Inflammasome activation consists of two stages: priming and activation [17]. The priming stage involves the transcriptional activation of inflammasome components and the proinflammatory cytokines pro-IL-1 and pro-IL-18, which is mediated by Toll-like receptors (TLRs) [17]. Priming causes inflammasome components to be synthesized and accumulated, preparing cells to react to later signals [17].

The inflammasome is activated when the sensor protein detects PAMPs or DAMPs [17]. Depending on the sensor protein and the type of input, the specific activation pathways might differ. For example, the NLRP3 inflammasome, one of the most thoroughly investigated inflammasomes, is activated by a variety of triggers, including microbial components, cholesterol crystals, extracellular adenosine triphosphate (ATP), reactive oxygen species (ROS), and lysosomal instability [18]. The precise processes behind NLRP3 activation are unknown, although they are believed to include ion fluxes, mitochondrial malfunction, and the synthesis of secondary messengers such as potassium efflux, calcium signaling, and the formation of oxidized mitochondrial DNA [18]. The NLRC4 inflammasome, on the other hand, is typically triggered by cytosolic bacterial flagellin or components of some bacteria’s type III secretion system (T3SS) [19]. Finally, the AIM2 inflammasome identifies pathogen or host cell cytosolic double-stranded DNA (dsDNA) [20]. AIM2 recruits ASC when it binds to dsDNA, resulting in the development of the inflammasome complex and the activation of caspase-1 [20].

## 4. Inflammasomes in Atherosclerosis

### 4.1. NLRP3 Inflammasome

**Figure 1:**
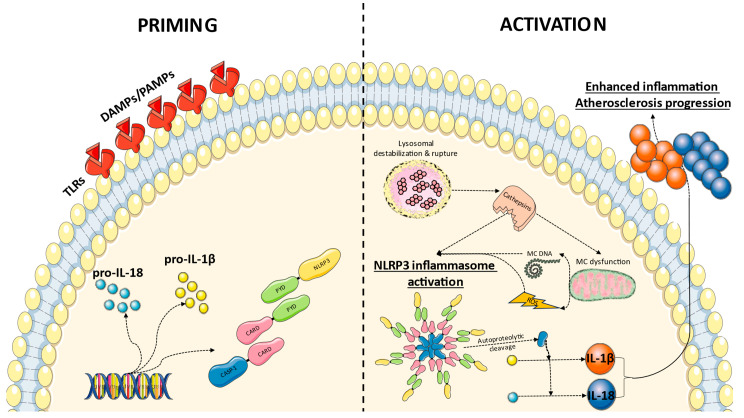
Proposed mechanism of NLRP3 inflammasome priming and activation in atherosclerosis. Recognition of DAMPs and PAMPs by toll-like receptors (TLRs) leads to the transcriptional activation of inflammasome components and the proinflammatory cytokines pro-interleukin(IL)-1 and pro-IL-18. During the activation phase, cholesterol crystals induce lysosomal destabilization and rupture, with the release of cathepsins. Those molecules promote NLRP3 inflammasome activation directly or indirectly through mitochondrial (MC) dysfunction and the release of mitochondrial DNA and reactive oxygen species (ROS). Following NLRP3 inflammasome formation, the autoproteolytic cleavage of caspase (CASP)-1 within the complex ensues and active CASP-1 ultimately cleaves pro-IL-1β and pro-IL-18 into their active forms, enhancing inflammation and atherosclerosis progression. CARD: caspase recruitment domain, PYD: pyrin domain.

The NLRP3 inflammasome, an upstream regulator of IL-1β production, has also been related to the atherosclerotic process. Cholesterol-crystal-induced NLRP3 inflammasome activation plays a pivotal role in the pathogenesis of atherosclerosis [21]. OxLDL, the key driver of inflammation in atherosclerosis, is responsible for the generation of cholesterol crystals within the plaques. Initially, macrophages engulf oxidized LDL particles, transforming into lipid-laden foam cells. As the plaque progresses, excessive cholesterol accumulation can lead to the formation of cholesterol crystals [21]. These cholesterol crystals are insoluble and can be highly inflammatory, leading to cellular stress and injury. The stressed and dying cells release DAMPs, including ATP, mitochondrial DNA, and ROS [21]. The cholesterol crystals and the released DAMPs serve as activators of the NLRP3 inflammasome [21]. However, the precise mechanism by which cholesterol crystals directly activate NLRP3 is still not fully understood. It is believed that cholesterol crystals are phagocytosed by macrophages and accumulate in lysosomes, disrupting the integrity of the lysosomal membrane and ultimately inducing lysosomal destabilization and rupture (Figure 1). Following lysosomal rupture, there is a release of lysosomal cathepsins, particularly cathepsin B [21]. These lysosomal cathepsins can then promote NLRP3 inflammasome activation [21]. Lysosomal cathepsins can directly cleave and activate the NLRP3 protein [22]. This cleavage can result in the exposure of specific domains within NLRP3 that are required for its activation. Lysosomal cathepsins can indirectly trigger mitochondrial dysfunction, which is a known contributor to NLRP3 inflammasome activation through ROS production and mitochondrial DNA release [22].

Following activation, NLRP3 recruits the adaptor protein ASC through homotypic interactions between the PYD of NLRP3 and the PYD of ASC [21]. ASC, in turn, recruits the effector pro-caspase-1 through homotypic CARD–CARD interactions [21]. The formation of the NLRP3 inflammasome complex leads to the autoproteolytic activation of caspase-1 within the complex [21]. Active caspase-1 then cleaves pro-IL-1β and pro-IL-18 into their active forms, IL-1β, and IL-18, respectively, which are then released from the cell [21]. These proinflammatory cytokines mediate the recruitment and activation of additional immune cells, further promoting the sustained inflammatory response within the plaque, with further recruitment of monocytes and T cells [23]. These cells further contribute to the formation of a necrotic core and thinning of the fibrous cap, rendering the plaque susceptible to rupture and thrombosis.

The NLRP3 inflammasome has been linked with various other deleterious pro-atherosclerotic conditions. To begin, it has been linked to diabetes-induced endothelial dysfunction, since NLRP3 knockdown in a diabetic atherosclerotic mice model led to decreased expression of adhesion molecules [24]. Nicotine intake can also influence plaque instability through the NLRP3 inflammasome due to the lysosomal dysfunction of VSMCs [25]. Moreover, in an angiotensin-II-induced hypertension mouse model, the NLRP3^-/-^ group had reduced blood pressure and largely recovered endothelial function and oxidative stress markers [26]. Additionally, hyperuricemia, a known risk factor of atherosclerotic cardiovascular disease, may promote atheroprone actions through the NLRP3 inflammasome-mediated endothelial cell pyroptosis [27].

Zheng et al. found that suppressing NLRP3 in ApoE^-/-^ mice fed a high-fat diet slowed plaque formation and increased proinflammatory cytokine production [28]. In the absence of NLRP3, the macrophage and lipid content of atherosclerotic plaques was reduced but the collagen content was increased, indicating a more stable phenotype [28]. Noticeably, NLRP3 signaling pathway molecules (NLRP3, ASC, caspase-1, IL-1β, and IL-18) were revealed to be overexpressed in atherosclerotic plaques from carotid artery specimens compared to non-atherosclerotic arteries [29,30]. Upregulation of ASC, caspase-1, and IL-18 was seen in coronary tree segments with advanced atherosclerosis, as well as the presence of NLRP3 inflammasome-positive foam cells around the necrotic core [31]. The NLRP3 inflammasome has been postulated as a mediator of VSMC phenotypic switch and incident plaque instability, as shown in an in vitro experimental study by Burger et al. [32]. In all, the findings mentioned above indicate the link between NRLP3 inflammasome and atherosclerosis. 

In clinical studies, we should initially state that smoking could be an important determinant of NLRP3 inflammasome activation, as displayed in the study of Mehta et al. who found a sevenfold higher expression of NLRP3 inflammasome markers in smokers with or without coronary artery disease (CAD) compared to the respective nonsmoking groups [33]. As far as plaque vulnerability is concerned, NLRP3 inflammasome expression was shown to be higher in patients with an ACS, followed by patients with stable angina, compared to those without coronary artery disease [34,35,36], showing a link between plaque vulnerability and clinical outcomes. These findings are in agreement with a recent study that demonstrated a significant correlation between vulnerable carotid atherosclerotic plaques and mRNA or protein expression of NLRP3 inflammasome components when compared to the stable plaque group [37]. Furthermore, the existence of cholesterol crystals in culprit lesions of patients with an acute coronary syndrome (ACS) was correlated with greater mRNA levels of NLRP3 in peripheral blood mononuclear cells, significantly higher concentrations of circulating IL-1β and IL-18, as well as a greater prevalence of thin-cap fibroatheromas, thrombus, plaque rupture, and spotty calcifications, among others, in optical coherence tomography [38]. Given the fact that NLRP3 mRNA was an independent predictor of cholesterol crystal occurrence, we can assume that cholesterol-crystal-induced NLRP3 inflammasome activation could represent an alarming feature of vulnerable plaques [38]. Adding to the above, the study of Komatsu et al. demonstrated that debris from 20 spontaneously ruptured aortic atherosclerotic plaques consistently contained cholesterol crystals that were often associated with the simultaneous presence of NLRP3, caspase-1, IL-1β, and IL-18 [39]. 

Genetic determinants of NLRP3 inflammasome expression may also influence clinical outcomes, with younger *rs10754555* G-allele carriers facing a lower risk of cardiovascular endpoints [40]. The role of this variant in NLRP3 gene expression was proven in the study of 538,167 subjects [41]. The carriers of the *rs10754555* variant had higher levels of CRP, while the carriers of the G-allele exhibited higher NLRP3 inflammasome activation in their isolated peripheral blood mononuclear cells. Furthermore, *rs10754555* carriers were characterized by a higher prevalence of CAD and greater incident mortality at follow-up, which was affected by NLRP3 inflammasome inducers such as urate, triglycerides, and apolipoprotein C3. Another allele that warrants consideration is the *rs10159239* A-allele, as shown by Akosile et al., who found an association between this variant and an increased coronary calcium score in Vietnam veterans [42].

### 4.2. Other Inflammasomes

Other inflammasomes have not been studied regarding their importance in the atherosclerotic process, with the exception of NLRP1 and NLRC4. In the study of 60 individuals with and without atherosclerotic lesions, the gene expression levels of NLRP1 and NLRC4 were significantly increased in subjects with atherosclerosis compared to healthy controls and were proportionate to stenosis severity [43]. As far as NLRP1 is concerned, the study of Bleda et al. showcased that, in the setting of increased triglycerides and very LDL (VLDL) concentrations, there is an increased expression of NLRP1 inflammasome in human arterial endothelial cells [44]. Such expression is associated with the severity of endothelial dysfunction [45].

## 5. Inflammasome Modulation: Therapeutic Perspectives

### 5.1. NLRP3 Inflammasome Modulation

#### 5.1.1. Anti-Atherosclerotic Approaches and NLRP3 Inflammasome

Beginning with statins, their impact in modulating NLRP3 inflammasome activation was initially shown in an experimental rat model of type 2 diabetes mellitus and diabetic cardiomyopathy. Rosuvastatin administration attenuated NRLP3 inflammasome components (NRLP3, ASC, pro-caspase-1, and caspase-1) expression, a fact that was accompanied by cardioprotective effects [46]. Similar findings were reported in a clinical study of patients with ACS treated with or without rosuvastatin [35]. Along those lines, atorvastatin also provided an anti-inflammatory effect through NLRP3 inflammasome inhibition in THP-1 cells stimulated with phorbol myristate acetate [47]. Another experimental study in endothelial cells proved that statins prevented oxLDL-induced NLRP3 inflammasome activation through a pregnane X receptor-dependent mechanism [48]. The combination of a statin with the ROS scavenger idebenone produced even more pronounced anti-atherosclerotic effects, evidenced by diminished plaque burden and enhanced plaque stability in ApoE^-/-^ mice [49]. This finding was accompanied by greater NLRP3 and IL-1β downregulation compared to each molecule alone [49]. Inhibition of proprotein convertase subtilisin-kexin type 9 (PCKS9) could provide additional NLRP3-inhibiting effects compared to other lipid-lowering drugs, as demonstrated in an observational study of 645 patients undergoing carotid endarterectomy [50]. Preclinically, treatment of hyperlipidemic ApoE^-/-^ mice with inclisiran attenuated atherosclerotic plaque formation by inhibiting pyroptosis with lower levels of cleaved caspase-1, NLRP3, ASC, Gasdermin D (GSDMD), and associated inflammatory cytokines (IL-1β and IL-18) [51].

Sodium-glucose cotransporter-2 (SGLT2) inhibitors have demonstrated anti-inflammatory properties [52,53], and may also influence the NLRP3 inflammasome pathway, as shown by Li et al., who demonstrated downregulated NLRP3/caspase-1 signaling following dapagliflozin administration in VSMCs, thus attenuating their pyroptosis [54]. 

Among antiplatelet pharmacotherapies, ticagrelor displayed NLRP3 inflammasome inhibitory actions in macrophages that were stimulated with lipopolysaccharide [55]. Critically, this action was independent of its effect on the P2Y_12_ receptor, since ticagrelor could specifically attenuate ASC complex formation. These findings were further verified in vivo and in patients after ACS. Aspirin also possesses NLRP3 inflammasome-inhibiting properties, since it protected microvascular endothelial cells stimulated with lipopolysaccharide from gap junction dysfunction by acting on this mediator [56].

Colchicine, an important anti-inflammatory molecule that has been effective in the prevention of major adverse cardiovascular events, has been suggested as an NLRP3 inflammasome inhibitor. Preclinically, the administration of colchicine in murine and human macrophages treated with oxidized LDL resulted in the inhibition of NLRP3 inflammasome formation [57]. Moving to clinical studies, Robertson et al. had initially proven that the administration of colchicine in patients with ACS resulted in lower expression of NLRP3 inflammasome markers (IL-1β, IL-18, pro-caspase-1, and caspase-1) from peripheral venous blood monocytes [58]. According to a biomarker substudy of the LoDoCo2 randomized clinical trial, extracellular vesicle NLRP3 protein levels were found decreased in subjects receiving colchicine compared to the placebo group, despite a lack of difference in serum NLRP3 protein levels [59]. These findings are not surprising since colchicine was previously proven to reduce transcoronary gradients of IL-1β, IL-6, and IL-18 in patients after an ACS [60].

The effects of exercise deserve an honorable mention. Obese mice on a high-fat diet exhibited a decrease in NLRP3 and caspase-1 gene and protein expression in their adipose tissue, accompanied by a lower expression of related cytokines (IL-1β and IL-18) in isolated bone-marrow-derived macrophages [61]. Li et al. found that implementation of aerobic exercise in hyperlipidemic ApoE^-/-^ mice reduced the expression of NLRP3 inflammasome-mediated pyroptosis in the aorta compared to the sedentary group and wild-type controls [62]. In a clinical trial, different exercise regimens were effective in reducing NLRP3 gene expression, verifying the potentially important role of this intervention against atherosclerosis [63]. The reduction in NLRP3 inflammasome-related circulating cytokines with exercise was further established in a recently published systematic review and meta-analysis of 19 randomized clinical trials, with aerobic exercise being the most effective modality [64].

#### 5.1.2. Direct NLRP3 Inflammasome Inhibitors

Direct pharmacological targeting of the NLRP3 inflammasome may be a viable strategy in the treatment of atherosclerotic disorders. MCC950 and VX765 are examples of experimental compounds exhibiting inhibitory characteristics. MCC950 prevents the oligomerization of NLRP3, which is a necessary step for the assembly of the inflammasome complex [65]. This compound was shown to reduce myocardial ischemia/reperfusion damage in a porcine model of myocardial infarction by decreasing neutrophil influx into the heart and IL-1 levels [66]. MCC950 then reduced maximum stenosis, average plaque size, and plaque volume in an ApoE^-/-^ animal model of carotid atherosclerosis, as well as macrophage accumulation and adhesion molecule mRNA expression [67]. Furthermore, infusion of platelet-derived extracellular vesicles containing MCC950 into ApoE^-/-^ mice prevented the development of atherosclerotic plaques by reducing inflammation, macrophage proliferation, and T cell localization in the plaque [68]. Sharma and colleagues conducted an interesting experiment in ApoE^-/-^ mice with streptozocin-induced diabetes [69]. In this harmful environment, NLRP3 inflammasome activation was more pronounced, although it was suppressed by MCC950 treatment. Furthermore, this molecule inhibited a variety of atherogenic processes, such as oxidative stress, inflammation, macrophage accumulation, necrotic core development, and fibrous cap thinning [69]. Zeng et al. demonstrated an improvement in macrophage pyroptosis and inflammation with MCC950 [70]. MCC950 was also shown to limit leukocyte migration by lowering endothelial cell chemokine and adhesion molecule expression [71]. Moving to VX765, it is a caspase-1 inhibitor since it irreversibly binds to the active site of caspase-1, preventing its enzymatic activity [72]. As a result, caspase-1 is unable to cleave pro-IL-1β and pro-IL-18 into their active forms. This molecule suppressed VSMC pyroptosis and atherosclerosis development in ApoE^-/-^ mice fed a western diet [73]. VX765 administration in vitro also abrogated NLRP3 inflammasome-mediated mitochondrial injury, while also enabling mitophagy, efferocytosis, and macrophage phenotypic switch toward an M2 phenotype [72]. Additionally, it prevented foam cell formation and macrophage pyroptosis. Importantly, when atherosclerotic Ldlr^-/-^ mice were treated with VX765, attenuated aortic atherosclerotic lesions were observed, along with a diminished necrotic core and macrophage infiltration compared to the control group [72]. Additionally, blood concentration of IL-1β was significantly reduced in VX765-treated mice [72]. Finally, tranilast, a tryptophan metabolite that suppresses NLRP3 inflammasome activation, was proven effective in reducing atherosclerotic lesion burden, macrophage content, and inflammatory molecule expression in atherosclerotic mouse models, accompanied by downregulated atherosclerotic plaque NLRP3 inflammasome expression due to enhanced ubiquitination [74].

While preclinical research on direct NLRP3 inflammasome inhibition is advancing, the first-in-man phase I trial of the GDC-2394, a small-molecule inhibitor of NLRP3, was unsuccessful. Its preclinical development in vitro and in vivo was uneventful, with an acceptable safety profile [75]. Despite favorable pharmacokinetic and pharmacodynamic characteristics, along with ex vivo inhibition of IL-1β and IL-18, there were important safety concerns due to the development of severe drug-induced liver injury [76]. This finding highlights the importance of continuous investigation on inflammasome modulation in order to unveil both the efficacy and, crucially, the safety of this approach. 

Other molecules with proven NLPR3 inflammasome-inhibiting effects, such as dapansutrile (OLT1177), INF39, CY-09, and JC124, among others, have not been studied in atherosclerotic experimental settings and their effectiveness in this field remains to be elucidated.

#### 5.1.3. Other Agents with Secondary NLRP3 Inflammasome-Modulatory Effects

Oridonin, a traditional Chinese herbal product, represents another potential therapeutic option against NLRP3 inflammasome activation according to the study of Wang et al. in a high-fat-fed ApoE^-/-^ mouse model [77]. Although not precisely clarified, oridonin may inhibit NLRP3 inflammasome activation by inhibiting its NLRP3 priming and assembly, as well as attenuating caspace-1 activity [78]. According to a recent study, NLRP3 RNA expression was also reduced in oridonin-treated rabbits that were on an atherogenic diet [79]. Moving to artesunate, a succinate derivative of artemisin that is utilized as an anti-malarial medication, it was shown to inhibit NLRP3 inflammasome activation in the arterial wall and macrophages of atherosclerotic rats, leading to a diminished release of proinflammatory cytokines [80]. Sinapine, a naturally occurring alkaloid, was found to protect against vascular endothelial dysfunction through inhibition of NLRP3 inflammation in an experimental hypertensive rat model [81]. Arglabin has received little attention in atherosclerosis research, although it has been shown to have anti-inflammatory (anti-inflammatory macrophage phenotypic switching) and hypolipidemic (reduction in total cholesterol and triglycerides) effects [82]. These were followed by a reduction in the growth of atherosclerotic lesions.

Modulating NLRP3 inflammasome activation against pyroptosis is another concept that is presently being explored in preclinical studies. Isoliquiritigenin, a licorice extract, attenuated TNF-α-induced NLRP3 inflammasome activation and pyroptosis in human umbilical vein endothelial cells by targeting sirtuin-6, which could be beneficial in the management of atherosclerosis [83]. Among the speculated mechanisms involved in this effect is the inhibition of NRLP3 inflammasome gene expression and assembly, as well as the suppression of pro-IL-1β and pro-IL-18 expression [78]. The management of NLRP3 inflammasome-mediated pyroptosis was the focus of the study conducted by Wang et al., who administered Pinocembrin in hyperlipidemic ApoE^-/-^ mice on a high-fat diet and observed the upregulation of nuclear factor erythroid 2-related factor 2/heme oxygenase-1 and downregulation of NLRP3 inflammasome-mediated pyroptosis [84]. Polydatin, a natural precursor of resveratrol, decreased NLRP3 protein expression, thus upregulating the process of autophagy and downregulating pyroptosis [85].

### 5.2. NLRP1 Inflammasome

While NLRP3 inflammasome remains the most extensively studied inflammasome, studies have also been conducted on other family members. Bleda et al., in an in vitro experiment involving individuals with peripheral arterial disease treated with or without simvastatin, found that the treatment of human arterial endothelial cells with plasma from peripheral arterial disease patients on simvastatin therapy could ameliorate NLRP1 inflammasome expression in the setting of peripheral arterial disease [86]. The modulation of Sterol regulatory element-binding protein 1 (SREBP-1) with statins could be the crucial step in this atheroprotective effect [87]. Moreover, the same research group confirmed the effectiveness of aspirin in inhibiting NLRP1 inflammasome [88]. Despite the encouraging evidence on the role and potential therapeutic implications of NLRP1 inflammasome, especially in peripheral arterial disease, studies of direct or indirect inhibitors are largely lacking, constituting an important knowledge gap.

### 5.3. AIM2 Inflammasome

At the preclinical level, few studies have attempted to modulate AIM2 inflammasome activation in an atherosclerotic context. Li et al. utilized the cyclic GMP-AMP (cGAMP) synthase (cGAS) antagonist A151 in a stroke mouse model and identified a significant neuroprotective effect through the reduction in infarct size, attenuation of cell death, and amelioration of the overall inflammatory response [89]. These findings were accompanied by AIM2 downregulation [89]. Moving to the study of Jiang et al. in experimental cerebral ischemia/reperfusion, the investigators noted that the long non-coding RNA (lncRNA) maternally expressed gene 3 (MEG3) positively regulates the expression of AIM2 via sponging miR-485, since this miR directly binds to 3′-UTR of AIM2 to dampen its expression [90]. This effect was also accompanied by decreased pyroptosis [90]. Other studies have reported similar findings in experimental cerebral ischemia models with the use of RGFP966, a histone deacetylase 3 inhibitor, and gonadal steroid hormones progesterone and 17β-estradiol [91,92]. Recently, a novel molecule named J114 demonstrated AIM2 inflammasome inhibitory effects through disturbance of the interaction of AIM2 with the adaptor protein ASC and inhibition of ASC oligomerization [93].

## 6. Clinical Implications and Conclusions

Inflammasomes have emerged as critical players in the pathophysiology of atherosclerosis, linking chronic inflammation to the development and progression of this prevalent cardiovascular disease. This review article has provided a comprehensive overview of the intricate roles played by inflammasomes in atherosclerosis, shedding light on their activation, molecular mechanisms, and their impact on plaque formation and rupture. The accumulated evidence underscores the significance of understanding inflammasome signaling as a potential avenue for therapeutic interventions in atherosclerosis.

From the presented literature, it is evident that dyslipidemia, a major risk factor for atherosclerosis, triggers inflammasome activation, particularly the NLRP3 inflammasome, through the recognition of lipid-related stimuli such as oxidized LDL and cholesterol crystals. The subsequent release of proinflammatory cytokines, such as IL-1β and IL-18, exacerbates the inflammatory milieu within the arterial walls, leading to the recruitment and differentiation of monocytes into foam cells and the formation of atherosclerotic plaques.

The interplay between inflammasome activation and atherosclerosis is a complex and multifaceted process involving a multitude of cell types and signaling pathways. Nonetheless, this intricate relationship offers potential therapeutic opportunities for the treatment of atherosclerosis. Inhibitors targeting inflammasome components, including NLRP3, have shown promise in preclinical studies and may represent a new class of anti-inflammatory agents capable of disrupting the inflammatory cascade underlying atherosclerosis.

As we delve further into understanding the crosstalk between inflammasomes and atherosclerosis, several key challenges and future directions arise. The identification of more specific and selective inflammasome inhibitors and the development of targeted delivery strategies hold great promise for mitigating off-target effects and enhancing therapeutic efficacy. Additionally, exploring the potential of lifestyle modifications and diet-induced changes to modulate inflammasome activation might provide complementary and preventive strategies for managing atherosclerosis and its cardiovascular consequences.

Several key questions also arise when considering inflammasome research in atherosclerosis. Differentiating the role of inflammasome activation in various cell types present in the atherosclerotic plaque is crucial, as this may variably influence atherosclerosis development and progression. The interaction of inflammasomes with other emerging factors of atherosclerosis such as the gut microbiome remains largely unexplored, while the genetic background of inflammasome activation and its role in atherosclerosis risk prognostication is another field that should be investigated further. It is also essential to note that the inflammasome research in atherosclerosis could be utilized and extended to other chronic diseases such as metabolic diseases, neurodegenerative diseases, cancer, and autoimmune diseases, among others.

Challenges may arise when translating inflammasome-targeted therapeutics from preclinical to human studies. First and foremost, while promising results in preclinical models may suggest efficacy, translating these findings into meaningful clinical outcomes in humans requires rigorous validation and understanding of the underlying disease mechanisms. Adding to this, the development of relevant human disease models for studying inflammasome-related diseases can be challenging. In vitro cell cultures and animal models might not fully recapitulate the complexity of human diseases, making it difficult to predict how therapies will perform in clinical settings. Furthermore, inflammatory diseases, such as atherosclerosis, are often multifaceted and can involve various cell types, signaling pathways, and molecular mechanisms, and targeting a single component, such as an inflammasome, might not be sufficient to address the complexity of the disease. Combinatorial therapies or broader immunomodulation strategies might be needed. It should also be stressed that human populations are genetically and clinically diverse and responses to inflammasome-targeted therapies can vary based on genetic factors, underlying health conditions, and other variables. Designing clinical trials that account for this heterogeneity is important for the accurate assessment of treatment effects. Moreover, modulating inflammasome activity can impact the immune response, potentially leading to unexpected side effects or compromising the organism’s ability to cope with infections. Careful evaluation of safety in humans is crucial to avoid unwanted immune suppression or other adverse effects. In this regard, determining the appropriate dosing regimen for inflammasome-targeted therapies can be complex, since the right balance between effective inhibition and potential adverse effects is critical. Moreover, the route of administration and pharmacokinetic properties of the therapeutic agent need to be considered. Last but certainly not least, the clinical relevance of inflammasome modulation remains to be unveiled.

In conclusion, this review consolidates the current knowledge on the involvement of inflammasomes in atherosclerosis and highlights their potential as targets for therapeutic intervention. With ongoing research and innovative discoveries, inflammasome-based therapies could pave the way for personalized and precise treatments for atherosclerosis, ultimately reducing the burden of cardiovascular disease and improving patient outcomes. Future investigations and collaborations should aim at unraveling the intricate mechanisms linking inflammasomes to atherosclerosis and driving translational breakthroughs toward effective treatments for this global health challenge.

## Data Availability

Data sharing is not applicable.
